# Phenotypic variation in familial chilblain lupus (FCL) and Aicardi-Goutières syndrome (AGS) associated with *TREX1* mutation in 4 family members

**DOI:** 10.1186/1546-0096-9-S1-P283

**Published:** 2011-09-14

**Authors:** James Glanville, Saleem Taibjee, Yanick Crow, Penny Davis, Clive Ryder, Taunton Southwood

**Affiliations:** 1 Department of Paediatric Rheumatology, Birmingham Children’s Hospital, UK; 2 Department of Dermatology, Birmingham Children’s Hospital, UK; 3 Department of Genetic Medicine, St Mary’s Hospital, UK

## Background

The spectrum of clinical manifestations associated with mutations of the *TREX1* gene (a major human 3’-5’ exonuclease which is an essential negative regulator of autoimmunity) includes systemic lupus erythematosus, FCL, AGS and retinal vasculopathy with cerebral leukodystrophy.

## Aim and Methods

To further define the clinical spectrum associated with *TREX1* mutations by reporting a series of cases from a single family.

## Results

The index case was a 10 year old Afro-Caribbean boy who initially presented with a non-progressive developmental delay and severe chilblains (the chilblains improved with methotrexate). He developed transient acute ataxia and flattened affect, with visual and auditory hallucinations. Parotid swelling, arthritis and proximal myopathy were noted, but autoantibodies were negative and complement studies normal. No viruses were detected in CSF or saliva. EEG was consistent with encephalopathy. Cerebral MRI was normal but basal ganglia calcification was seen on CT. Skin histology was consistent with FCL.

His mother and 2 siblings (3 year old brother and 6 year old sister) also had chilblains (each child had a different father). No other family member had developmental delay, other evidence of autoimmunity or inherited metabolic disease. Neither sibling had CT evidence of basal ganglia calcification. All 4 affected family members had heterozygote mutation of the *TREX1* gene (p.Asp18Asn).

## Conclusion

FCL is a genodermatosis characterised by painful bluish-red skin lesions. AGS is a progressive, chronic encephalopathy associated with basal ganglia calcification. FCL and AGS are proposed to be different clinical manifestations associated with a variety of *TREX1* mutations. Our cases demonstrate the variation in clinical phenotype associated with a single *TREX1* mutation, ranging from typical FCL to acute encephalopathy with features suggestive of AGS, in 4 family members.

